# Characterization of the complete mitochondrial DNA of *Theretra japonica* and its phylogenetic position within the Sphingidae (Lepidoptera, Sphingidae)

**DOI:** 10.3897/zookeys.754.23404

**Published:** 2018-05-03

**Authors:** Jun Li, Rui-Rui Lin, Yao-Yao Zhang, Kun-Jie Hu, Ya-Qi Zhao, Yan Li, Zhuo-Ran Huang, Xu Zhang, Xue-Xia Geng, Jian-Hua Ding

**Affiliations:** 1 School of Life Sciences, Huaibei Normal University, Huaibei, China

**Keywords:** Lepidoptera, mitogenome, Sphingidae, *Theretra japonica*

## Abstract

In the present study, the complete mitogenome of *Theretra japonica* was sequenced and compared with other sequenced mitogenomes of Sphingidae species. The mitogenome of *T. japonica*, containing 37 genes (13 protein-coding genes, 22 tRNA genes, and two rRNA genes) and a region rich in adenine and thymine (AT-rich region), is a circular molecule with 15,399 base pairs (bp) in length. The order and orientation of the genes in the mitogenome are similar to those of other sequenced mitogenomes of Sphingidae species. All 13 protein-coding genes (PCGs) are initiated by ATN codons except for the cytochrome *C* oxidase subunit 1 gene (*cox1*) which is initiated by the codon CGA as observed in other lepidopteran insects. Cytochrome *C* oxidase subunit 2 gene (*cox2*) has the incomplete termination codon T and NADH dehydrogenase subunit 1 gene (*nad1*) terminates with TAG while the remainder terminates with TAA. Additionally, the codon distributions of the 13 PCGs revealed that Ile and Leu2 are the most frequently used codon families and codons CGG, CGC, CCG, CAG, and AGG are absent. The 431 bp AT-rich region includes the motif ATAGA followed by a 23 bp poly-T stretch, short tandem repeats (STRs) of TC and TA, two copies of a 28 bp repeat ‘ATTAAATTAATAAATTAA TATATTAATA’ and a poly-A element. Phylogenetic analyses within Sphingidae confirmed that *T. japonica* belongs to the Macroglossinae and showed that the phylogenetic relationship of *T. japonica* is closer to *Ampelophaga rubiginosa* than *Daphnis nerii*. Phylogenetic analyses within *Theretra* demonstrate that *T. japonica*, *T. jugurtha*, *T. suffusa*, and *T. capensis* are clustered into one clade.

## Introduction

The Sphingidae (Lepidoptera) moths are commonly known as hawk moths, sphinx moths, or hornworms and include 1,463 species (Nieukerken et al. 2011). *Theretra japonica*, known as a pest, is widely distributed in Korea, Japan, Russia, and China. Its larva eats leaves and is harmful to many important ornamental plants, such as *Vitis vinifera*, *Saxifraga stolonifera*, *Hoya carnosa*, and *Cayratia japonica* etc. (Zhu and Wang 1997; Shirotsuka and Yano 2012).

Mitochondrial DNA sequences have been widely used to study the molecular evolution of insects due to protein-coding genes (PCGs) sequence conservatism, maternal inheritance, and rapid evolution (Cameron 2014). In Sphingidae, however, only the complete mitochondrial DNA sequences of *Notonagemia analis* (KU934302) (Kim et al. 2016), *Sphinx morio* (KC470083) (Kim et al. 2013), *Manduca sexta* (EU286785) (Cameron and Whiting 2008), *Ampelophaga rubiginosa* (KT153024) (Xin et al. 2017), *Agrius convolvuli* (https://doi.org/10.1139/gen-2016-0058) (Dai et al. 2017), and *Daphnis nerii* (https://doi.org/10.1371/journal.pone.0178773.s001) (Sun et al. 2017) have been reported up to now. Among these six species, *N. analis*, *S. morio, A. convolvuli*, and *M. sexta* belong to the subfamily Sphinginae, while *A. rubiginosa* and *D. nerii* belong to the Macroglossinae. More mitogenome sequences from Sphingidae will be helpful to discover the interfamilial phylogenetic relationships. *Theretra japonica* is taxonomically classified into the subfamily Macroglossinae according to its morphology (Zhu and Wang 1997), but its mitogenome has not yet been reported, nor a phylogenetic analysis based on this.

In this study polymerase chain reaction (PCR) amplification, DNA sequencing, and overlapped fragments assembling methods were used to determine the complete mitogenome of *T. japonica*. The characteristics of the mitogenome were also analyzed and a phylogeny was constructed. These will be helpful to understand the evolutionary position of *T. japonica* within Sphingidae.

## Materials and methods

### Specimens sampling and DNA extraction

The specimen was collected from Xiangshan mountain, Huaibei city, Anhui province, China (33°59.02'N, 116°48.57'E), and then was preserved in -20 °C refrigerator. Total genomic DNA was extracted from the abdomen of the moth (voucher number TJ20171011) using Ezup Column Animal Genomic DNA Purification Kit (Sangon Biotech, China) following the manufacturer’s instructions. The extracted DNA samples were stored at -20°C. The specimen and the template DNA are respectively deposited in Specimens Room within the Human and Animal Genetics Laboratory, School of Life Sciences, Huaibei Normal University.

### PCR amplification and DNA sequencing

The mitochondrial DNA fragments were amplified by PCR method and the total genomic DNAs were used as template. PCR primers were designed according to the conservative sequences of mitochondrial DNA of Lepidoptera insects and showed in Table 1. The overlapping fragments were amplified using PrimeSTAR® GXL DNA Polymerase (Takara, China) according to the manufacturer’s instructions. PCR reaction mixture (25 μL in total) included 5 μL 5× PrimeSTAR GXL Buffer, 2 μL dNTP mixture (2.5 mM each), 2.5 μL primer (10 μM) each, 1 μL PrimeSTAR GXL DNA Polymerase, 1 μL template DNA (100 ng/μL) and 11 μL double distillated water. PCR reaction was performed in Eppendoff Mastercycler gradient PCR instrument under the following conditions: 30 sec at 98 °C; followed by 30 cycles of 15 sec at 98 °C, 15 sec at 40–55 °C and 2–8 min at 68 °C; and at last 10 min at 68 °C. PCR productions were confirmed by 1% (w/v) agarose gel electrophoresis and sequenced at least three times.

**Table 1.** Details of the primers used to amplify the mitochondrial DNA of *T. japonica*.

**Primer name**	**Orientation**	**Annealing position (bp)**	**Nucleotide sequence (5'-3')**	**PCR length**
Q1F	F	1314-1336	AAACTAATAATCTTCAAAATTAT	
Q1R	R	6236-6213	AATATTAATGGAATTTAACCACTA	4923
Q2F	F	6193-6216	TAAGCTGCTAACTTAATTTTTAGT	
Q2R	R	9637-9617	GTTTCAATAATCCGAACTCAT	3445
Q3F	F	8601-8618	CGTCTATGCAATCGCTCA	
Q3R	R	12319-12302	GCATTACTTGGAGGGTTG	3719
Q4F	F	11600-11620	TCCCTATGTTATTACAGGACA	
Q4R	R	14809-14791	CCAGCAGTTGCGGTTATAC	3210
Q5F	F	14637-14659	TAATAGGGTATCTAATCCTAGTT	
Q5R	R	1400-1378	ATATAAAATTGCAAATTTTAAGG	2163

### Sequences assembly, annotation and analysis

The overlapping fragments were assembled into a complete linear mitochondria DNA sequence using the DNAStar package (DNAStar Inc. Madison, WI, USA), and the mitogenome was annotated using MITOS2 (Bernt et al. 2013). The PCGs and ribosomal RNA (rRNA) genes were verified by NCBI BLAST. The transfer RNA (tRNA) genes were verified by tRNAscan-SE2.0 (Lowe and Chan 2016; Lowe and Eddy 1997). Barcoding analysis was performed in Bold Systems v4 using *cox1* as the marker following the recommendations of Botera-Castro et al (2016). The protein sequences were translated with the invertebrate mitochondrial genetic code. Nucleotides composition and codon usage were counted using MEGA 7.0. The bias of nucleotide composition was measured as AT skew (AT skew = (A-T)/(A+T)) and GC skew (GC skew = (G-C)/(G+C)) respectively.

### Phylogenetic analyses

To clarify the phylogenetic position of *T. japonica* within the Sphingidae, all published complete mitogenomes of members of the Sphingidae were collected and their 13-protein amino acid (AA) sequences were incorporated together for alignment and phylogenetic tree construction. Sequences were aligned using ClustalX 2.1 (Larkin et al. 2007) and phylogenetic trees were constructed using the Neighbor-Joining (NJ) and Maximum likelihood analysis (ML) methods with bootstrap test of 1000 replications by MEGA 7.0 (Kumar et al. 2016). *Bombyx mori* (Bombycidae, AF149768) and *Antheraea pernyi* (Saturniidae, AY242996) were utilized as outgroups. The gaps or missing data subsets were completely deleted. Before constructing the ML phylogeny, Mega 7.0 was utilized to find the best model (mtREV + F + G). The NJ phylogeny was constructed using Poisson model and bootstrap for 1000 times. The parameter of Rates among Sites was set as Gamma distributed (G) and value as 13. The parameter for Pattern among Lineages was set as homogeneous.

The *cox1* barcodes (481 barcodes) were gathered for the genus *Theretra* in BOLD system v4 to construct phylogeny. Those barcodes without gaps or missing nucleotides (total 658 bp in size) were selected to construct ML phylogenetic tree, and finally 285 barcodes (41 species) were utilized. The *cox1* sequence of *B. mori* (AF149768) was used as outgroup. The best model was GTR + G + I. To infer nodal support, bootstrapping was conducted 1000 times.

## Results and discussion

### Genome organization and nucleotides composition

**Figure 1.**
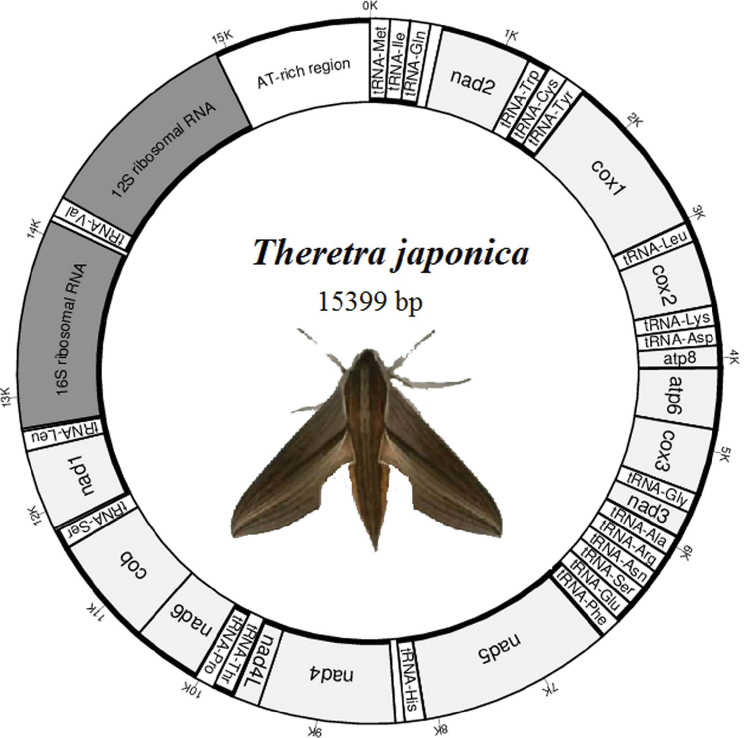
The schematic illustration for mitogenome of *T. japonica*. Gene order and positions are shown. *cox1*, *cox2*, and *cox3* refer to the cytochrome *c* oxidase subunits; *cob* refers to cytochrome *b*; *nad1-nad6* refers to NADH dehydrogenase components; *rrnL* and *rrnS* refer to ribosomal RNAs. The bold lines on outer or inner ring represent that the genes lie in the majority-coding strand (J-strand) or the minority-coding strand (N-strand).

The complete mitogenome sequence of *T. japonica* (MG655620) is 15,399 base pairs (bp) in length, shorter than *M. sexta* but longer than the other 5 species of Sphingidae. It contains 13 PCGs, 22 tRNAs genes, two rRNAs genes, and an AT-rich region with a length of 431 bp (Fig. 1, Suppl. material 2). Among the 37 genes, 23 genes are encoded by the majority-coding strand (J-strand) and 14 genes are encoded by the minority-coding strand (N-strand). The gene order and orientation are consistent to the other Sphingidae species.

The nucleotide composition in J-strand of *T. japonica* mitogenome is as follows: 6,331 bp (41.11%) A, 6,043 bp (39.24%) T, 1,883 (12.23%) C, and 1,142 bp (7.42%) for G. A+T accounts for 80.36%, which is slightly higher than *D. nerii* (80.29%) but lower than *M. sexta* (81.79%), *S. morio* (81.17%), *N. analis* (81.79%), *A. convolvuli* (81.49%), and *A. rubiginosa* (81.5%) (Sun et al. 2017; Xin et al. 2017; Dai et al. 2016). The AT skew and GC skew of *T. japonica* J-strand are 0.023 and -0.245 respectively (Table 2). The GC skews of the seven Sphingidae species are all negative and the AT skews are positive except for *M. sexta* (-0.005) and *A. convolvuli* (-0.001) (Sun et al. 2017; Xin et al. 2017; Dai et al. 2016).

In the mitogenome of *T. japonica*, there are 13 gene overlaps and 15 intergenic spacers (Suppl. material 2). The 13 gene overlaps range from 1 to 17 bp in size and the longest is present between *trnF* and *nad5*. The 15 intergenic spacers range from 1 to 88 bp in size, and the longest is present between *trnQ* and *nad2*, which is also founded in the other six Sphingidae species. However, the intergenic spacer between *trnQ* and *nad2* of *T. japonica* is longer than that of the other six species, which range from 51bp in *N. analis* to 56 bp in *A. rubiginosa*.

**Table 2.** Base composition of protein-coding, tRNA and rRNA genes, and A+T rich region of *T. japonica* mitogenome.

**Genes or regions**	**Size (bp)**	**Base composition (%)**				**A+T (%)**	**AT skewness**	**GC skewness**
		**A**	**T**	**C**	**G**			
*nad2*	1014	37.87	47.14	9.66	5.33	85.01	-0.109	-0.289
*cox1*	1536	32.42	38.61	15.63	13.35	71.03	-0.087	-0.079
*cox2*	685	37.96	39.27	13.14	9.64	77.23	-0.017	-0.154
*atp8*	165	44.85	44.24	9.09	1.82	89.09	0.007	-0.667
*atp6*	678	36.28	41.89	14.16	7.67	78.17	-0.072	-0.297
*cox3*	792	34.22	39.52	14.52	11.74	73.74	-0.072	-0.106
*nad3*	354	36.44	43.79	12.99	6.78	80.23	-0.092	-0.314
*nad5*	1758	32.82	48.81	5.92	12.46	81.63	-0.196	0.356
*nad4*	1332	33.63	48.42	6.38	11.56	82.06	-0.180	0.289
*nad4L*	291	30.93	52.58	3.78	12.71	83.06	-0.259	0.542
*nad6*	531	40.49	45.20	8.66	5.65	85.69	-0.056	-0.210
*cob*	1149	34.64	40.82	14.45	10.10	75.46	-0.082	-0.177
*nad1*	936	30.02	48.08	7.48	14.42	78.10	-0.231	0.317
Total	11221	34.50	44.38	10.53	10.59	78.88	-0.125	0.003
*tRNA*	1465	41.77	39.32	8.05	10.85	81.09	0.030	0.148
*rRNA*	2048	41.50	42.24	5.08	11.18	83.74	-0.009	0.375
AT-rich region	431	41.50	42.24	5.08	11.18	93.04	-0.007	-0.400
Complete mitogenome	15399	41.11	39.24	12.23	7.42	80.36	0.023	-0.245

### Protein-coding genes and codon usage

The *T. japonica* mitogenome contains 13 PCGs as expected with a total of 11,221 bp in size. All the PCGs are initiated with ATN codons, except for *cox1*, which uses CGA as the initiation codon. Most PCGs are terminated with TAA codon while *nad1* uses TGA as termination codon. And yet, *cox2* has an incomplete termination codon ‘T’. The incomplete termination codon ‘T’ or ‘TA’ could become TAA by posttranscriptional polyadenylation (Ojala et al. 1981). In the 13 PCGs, only the AT skewness of *atp8* is positive (0.007) while the others are negative. The GC skewness of *nad1*, *nad4*, *nad4L*, and *nad5* are positive and all lie in the N-strand.

**Figure 2.**
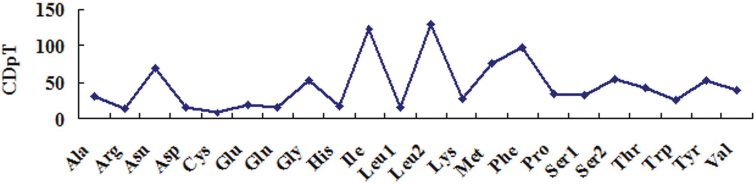
The amino acids usage in the mitogenome of *T. japonica*. CDpT = codons per thousand codons.

**Figure 3.**
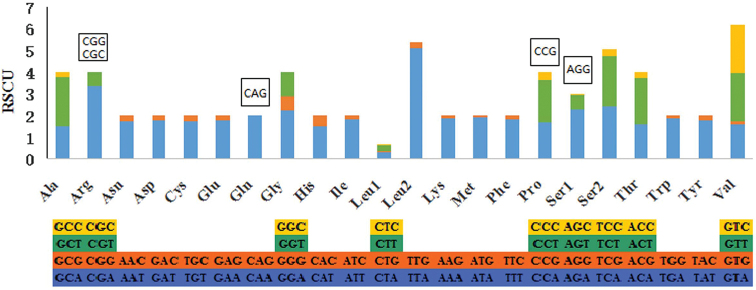
The relative synonymous codon usage (RSCU) in the mitogenome of *T. japonica*. The codons listed up the columns are absent in *T. japonica*.

The amino acids (AAs) components and their codon usage in the PCGs of *T. japonica* mitogenome were also analyzed. The results reveal that two codon families (Ile and Leu2) are more than 100 codons per thousand codons (CDpT), six codon families (Asn, Gly, Met, Phe, Ser2, and Tyr) are between 50 CDpT and 100 CDpT, and the other fourteen codon families are less than 50 CDpT (Fig. 2). AAs codon usage is assessed by values of the relative synonymous codon usage (RSCU) and five codons (CGG, CGC, CCG, CAG, and AGG) are absent in the PCGs of *T. japonica* (Fig. 3). It is found that the codons with high G and C content are likely not to be favored in lepidopteran insects (Dai et al. 2016).

### Ribosomal RNA genes and transfer RNA genes.

**Figure 4.**
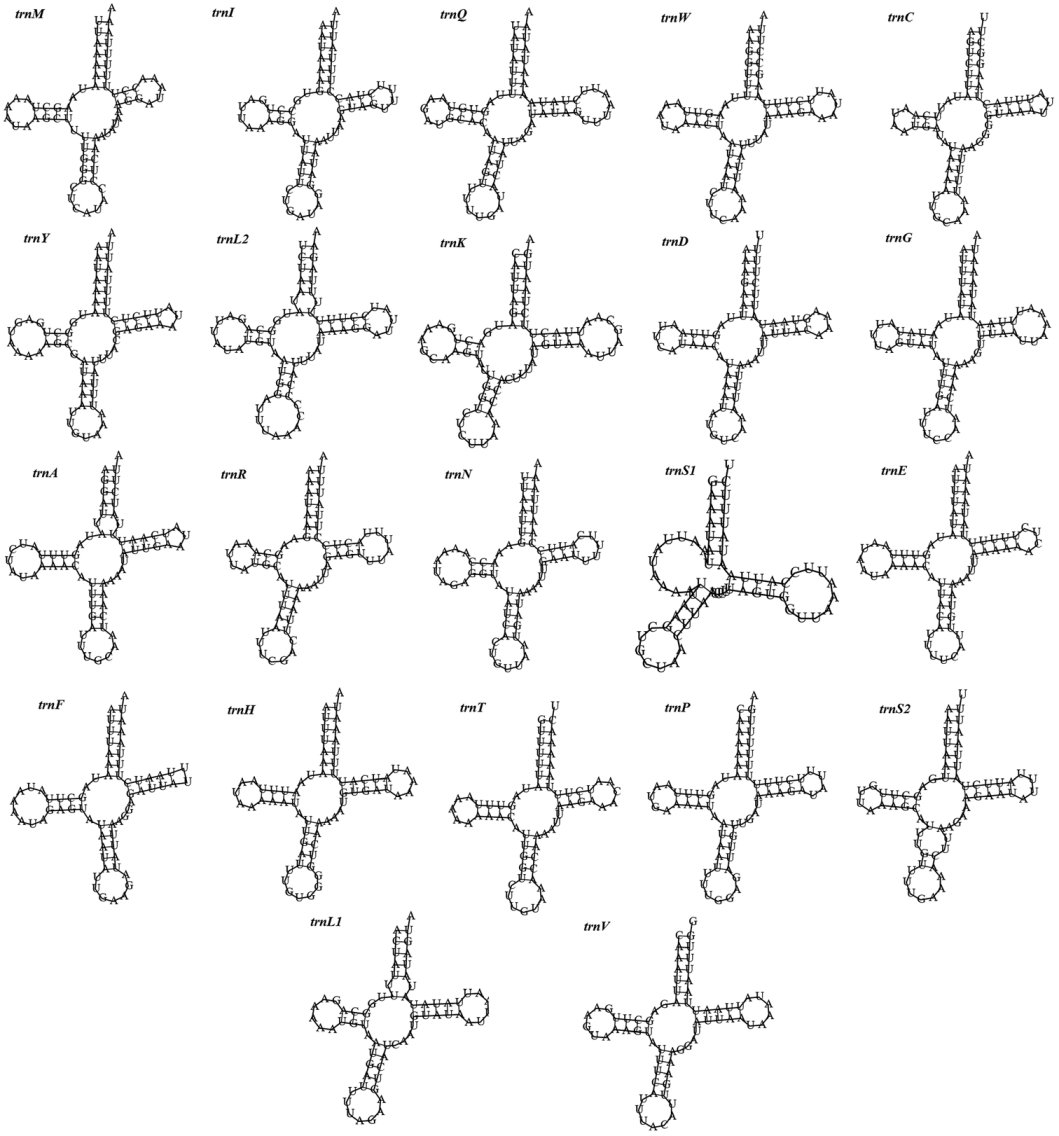
The cloverleaf secondary structure of transfer RNA of *T. japonica*.

The 22 rRNA genes of *T. japonica* range from 64 bp to 71 bp in size and totally comprise 1,465 bp of the whole mitogenome. Of these genes, 14 are encoded in J-strand and 8 in N-strand just as other Sphingidae moths. The predicted secondary structures of the tRNAs are shown in Figure 4. All the tRNA genes except for *trnS1* could be folded into the typical cloverleaf secondary structure (Fig. 4). The A+T content of the 22 tRNAs is 83.74%, with a positive AT skew (0.030) and GC skew (0.148).

Just as the other Sphingidae species there are two rRNA genes in *T. japonica* with a total length of 2,048 bp. The large ribosomal gene (*rrnL*) locates between *trnL1* and *trnV*, with a length of 1,284 bp whereas the small ribosomal gene (*rrnS*) locates between *trnV* and the A+T-rich region with a length of 764 bp. The AT skew is slightly negative (−0.009), but the GC skew is strongly positive (0.375). The total A+T content of the rRNA genes (83.74%) is higher than that of total tRNA genes (81.09%) and total PCG genes (79.10%).

### A+T rich region

**Figure 5.**
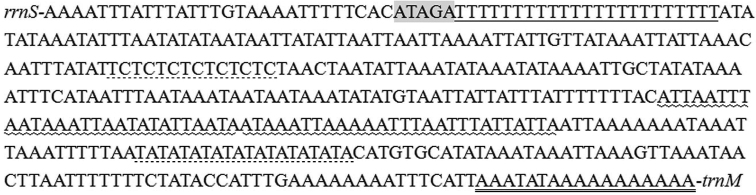
Features of the A+T-rich region of *T. japonica*. The ATATG motif is shaded. The polyT stretch is underlined while the poly-A stretch is double underlined. The TA and GC repeats sequence are indicated by dotted underlining. The 28 bp repeats ‘ATTAAATTAATAAATTAATATATTAATA’ are labeled with wave underlining.

The A+T rich region locates between *rrnS* and *trnM* in *T. japonica* with 431 bp in length and serves as the initiation of mitochondrial replication in both vertebrates and invertebrates (Cameron 2014). This region contains the highest A+T content (93.04%) in the mitogenome of *T. japonica*. As the other lepidopteran mitogenomes, the A+T rich region of *T. japonica* has some conserved structures including the motif ‘ATAGA’ followed by a 23 bp poly-T stretch, a short tandem repeats (STRs) of TC and TA, two copies of a 28 bp repeat ‘ATTAAATTAATAAATTAATATATTAATA’, and a poly-A element upstream of the *trnM* (Fig. 5). The poly-T element may be associated with mitochondrial transcription controlling and replication initiation.

### Barcoding analysis and phylogenetic analysis

**Figure 6.**
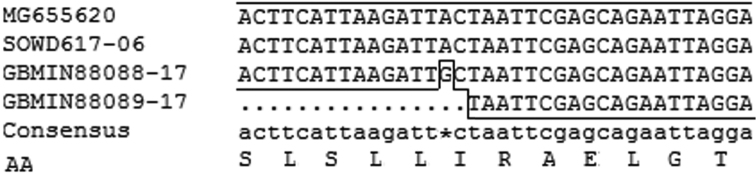
Barcoding analysis of *T. japonica*.

By using the identified *cox1* barcode and aligning it with the three barcodes of *T. japonica* deposited in Bold system v4 we found that the similarities are 100% with GBMIN88089-17 (KX440686, 541 bp) and SOWD617-06 (JN678612, 658 bp), and 99.85% with GBMIN88088-17 (KT988392, 658 bp) respectively. The only mutation of GBMIN88088-17 takes place at the third position nucleotide of a codon for Leucine which mutates from TTA to TTG, but the mutation is synonymous because it does not change its AA code (Fig. 6). SOWD617-06 was collected from Sichuan province, China (Wilson et al. 2011) and the positions where the two others were collected are unspecified.

**Figure 7.**
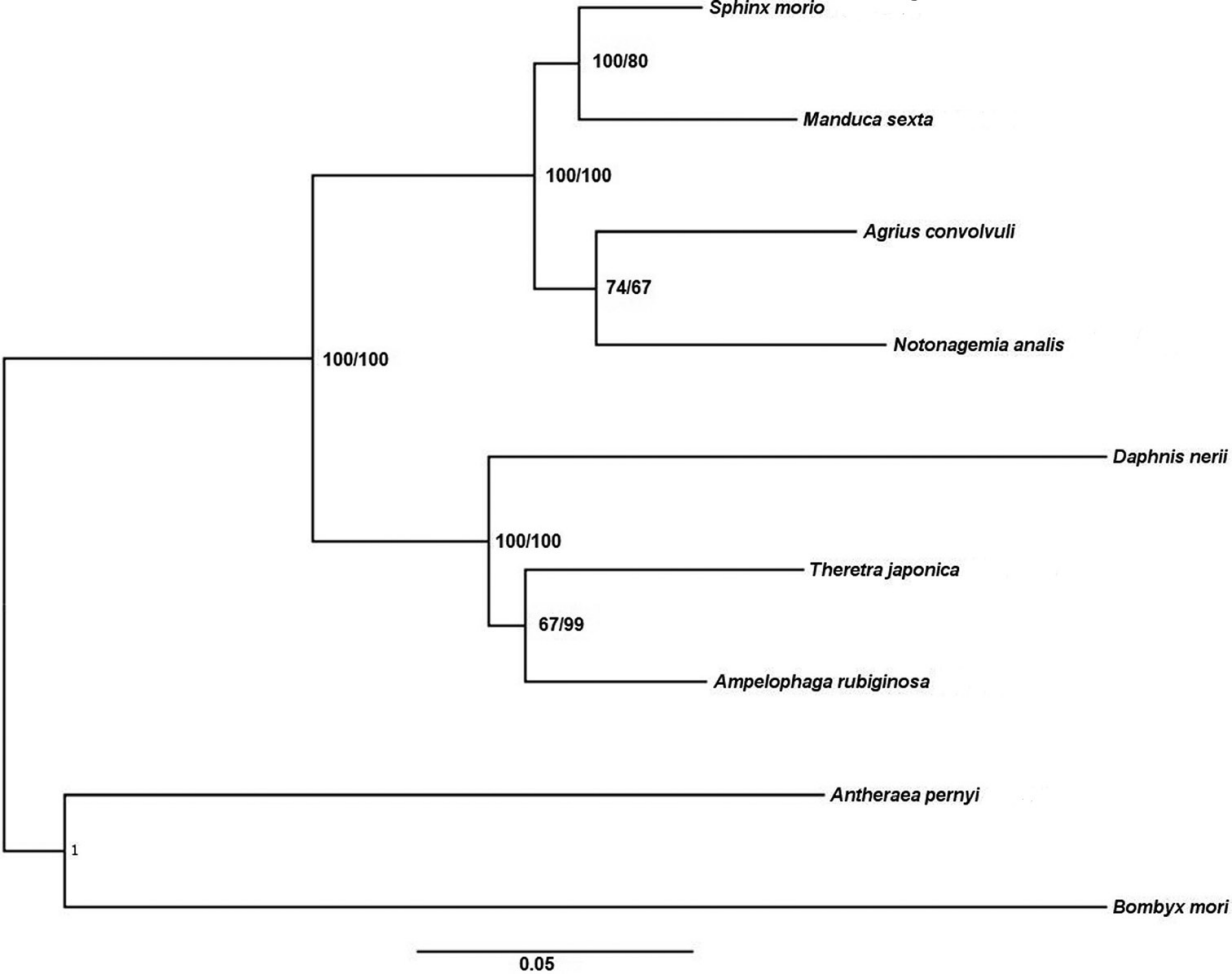
Phylogenetic analysis. Phylogenetic tree constructed using NJ and ML methods based on the amino acid sequences of 13 PCGs of 7 species with *Bombyx mori* (Lepidoptera: Bombycidae) and *Antheraea pernyi* (Lepidoptera: Saturniidae) as outgroups. The support values at the nodes represent bootstrap values for NJ and ML respectively.

Phylogenetic analyses were firstly based on the sequences of 13 PCGs of seven mitogenomes using NJ and ML Methods. The phylogenetic trees constructed by NJ and ML are consistent with high intermediate bootstrap values (Fig. 7). The Sphingidae includes three subfamilies, Sphinginae, Macroglossinae, and Smerinthinae, but no complete mitogenome from the Smerinthinae has been published so far. Among these species of Sphingidae, *T. japonica*, *A. rubiginosa* and *D. nerii* belong to Macroglossinae and the other four belong to Sphinginae. *T. japonica, D. nerii* and *A. rubiginosa* are clustered together into a monophyletic group and *T. japonica* is phylogenetically closer to *A. rubiginosa* than *D. nerii*. The phylogenetic tree of these seven species is ((*T. japonica* + *A. rubiginosa*) + *D. nerii*) + ((*N. analis + A. convolvuli*) + (*S. morio* + *M. sexta*)) which is also partly supported by Kawahara et al (2009). ML phylogenetic tree constructed for genus *Theretra* demonstrates that *T. japonica*, *T. jugurtha*, *T. suffusa*, and *T. capensis* are clustered into one clade (Suppl. material 1).

## Supplementary materials

Supplementary material 1 Phylogenetic analysis Data type: molecular data Explanation note: ML tree constructed based on the *cox1* barcodes of genus *Theretra* using *B. mori* as outgroups. The asterisk represents the species researched presently. This is an open access article distributed under the terms of the Creative Commons Attribution License (CC BY 4.0), which permits unrestricted use, distribution, and reproduction in any medium, provided the original author and source are credited.Li J, Lin R-R, Zhang Y-Y, Hu K-J, Zhao Y-Q, Li Y, Huang Z-R, Zhang X, Geng X-X, Ding J-H

Supplementary material 2 List of annotated mitochondrial genes of *T. japonica* Data type: molecular data This is an open access article distributed under the terms of the Creative Commons Attribution License (CC BY 4.0), which permits unrestricted use, distribution, and reproduction in any medium, provided the original author and source are credited.Li J, Lin R-R, Zhang Y-Y, Hu K-J, Zhao Y-Q, Li Y, Huang Z-R, Zhang X, Geng X-X, Ding J-H
