# Supplementary figures and images for: Characterization of the complete mitochondrial DNA of Theretra japonica and its phylogenetic position within the Sphingidae (Lepidoptera, Sphingidae)
Source: Zookeys. 2018 May 3;(754):127–39. doi: 10.3897/zookeys.754.23404 (PMC5945705; doi:10.3897/zookeys.754.23404)

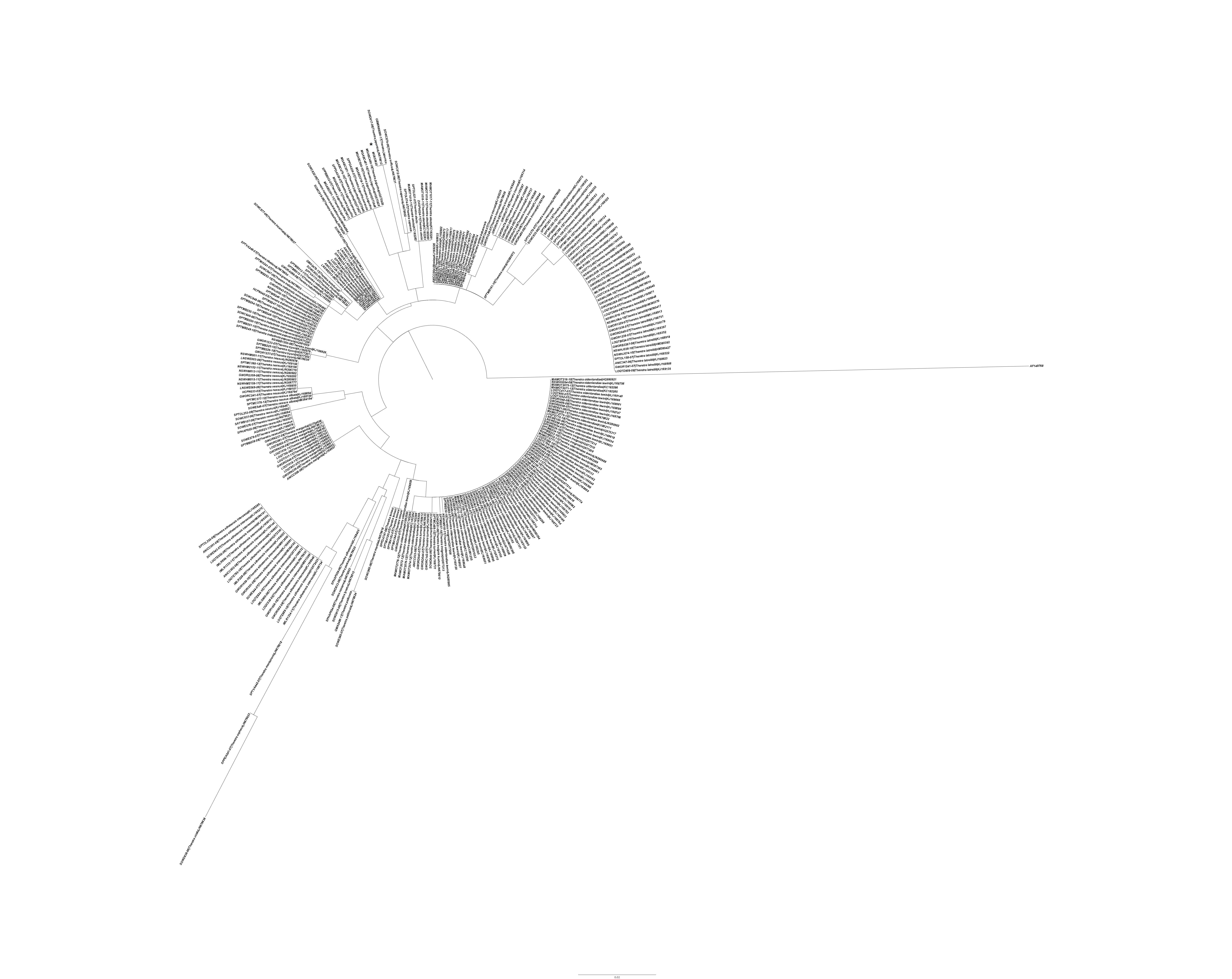

Supplement: Supplementary material 1 — Phylogenetic analysis [file zookeys-754-127-s001.jpg]
