# Supplementary material for: Characterization of the complete mitochondrial DNA of Theretra japonica and its phylogenetic position within the Sphingidae (Lepidoptera, Sphingidae)
Source: Zookeys. 2018 May 3;(754):127–39. doi: 10.3897/zookeys.754.23404 (PMC5945705; doi:10.3897/zookeys.754.23404)
Supplement: Supplementary material 2 — List of annotated mitochondrial genes of T. japonica [file zookeys-754-127-s002.docx]

**Supplementary material: 1 Table 1.** List of annotated mitochondrial genes of *T. japonica.*

| **Gene name** | **Start** | **Stop** | **Strand** | **Length** | **Anti-**  **codon** | **Start codon** | **End codon** | **Intergenic**  **nucleotides** |
| --- | --- | --- | --- | --- | --- | --- | --- | --- |
| *trnM* | 1 | 66 | + | 66 | CAT | / | / | 0 |
| *trnI* | 67 | 130 | + | 64 | GAT | / | / | -3 |
| *trnQ* | 128 | 196 | - | 69 | TTG | / | / | 88 |
| *nad2* | 285 | 1298 | + | 1014 | / | ATT | TAA | -2 |
| *trnW* | 1297 | 1363 | + | 67 | TCA | / | / | -8 |
| *trnC* | 1356 | 1419 | - | 64 | GCA | / | / | 0 |
| *trnY* | 1420 | 1485 | - | 66 | GTA | / | / | 10 |
| *cox1* | 1496 | 3031 | + | 1536 | / | CGA | TAA | -5 |
| *trnL2* | 3027 | 3093 | + | 67 | TAA | / | / | 0 |
| *cox2* | 3094 | 3778 | + | 685 | / | ATG | T | -3 |
| *trnK* | 3776 | 3846 | + | 71 | CTT | / | / | -1 |
| *trnD* | 3846 | 3911 | + | 66 | GTC | / | / | 0 |
| *atp8* | 3912 | 4076 | + | 165 | / | ATC | TAA | -7 |
| *atp6* | 4070 | 4747 | + | 678 | / | ATG | TAA | 2 |
| *cox3* | 4750 | 5541 | + | 792 | / | ATG | TAA | 2 |
| *trnG* | 5544 | 5610 | + | 67 | TCC | / | / | 0 |
| *nad3* | 5611 | 5964 | + | 354 | / | ATT | TAA | 7 |
| *trnA* | 5972 | 6038 | + | 67 | TGC | / | / | 3 |
| *trnR* | 6042 | 6106 | + | 65 | TCG | / | / | 1 |
| *trnN* | 6108 | 6174 | + | 67 | GTT | / | / | 0 |
| *trnS1* | 6175 | 6239 | + | 65 | GCT | / | / | 3 |
| *trnE* | 6243 | 6310 | + | 68 | TTC | / | / | -2 |
| *trnF* | 6309 | 6375 | - | 67 | GAA | / | / | -17 |
| *nad5* | 6359 | 8116 | - | 1758 | / | ATT | TAA | -3 |
| *trnH* | 8114 | 8180 | - | 67 | GTG | / | / | 68 |
| *nad4* | 8249 | 9580 | - | 1332 | / | ATG | TAA | 0 |
| *nad4l* | 9581 | 9871 | - | 291 | / | ATG | TAA | 14 |
| *trnT* | 9886 | 9950 | + | 65 | TGT | / | / | -1 |
| *trnP* | 9950 | 10015 | - | 66 | TGG | / | / | 8 |
| *nad6* | 10024 | 10554 | + | 531 | / | ATT | TAA | -1 |
| *cob* | 10554 | 11702 | + | 1149 | / | ATG | TAA | -2 |
| *trnS2* | 11701 | 11765 | + | 65 | TGA | / | / | 20 |
| *nad1* | 11786 | 12721 | - | 936 | / | ATG | TAG | 1 |
| *trnL1* | 12723 | 12789 | - | 67 | TAG | / | / | 18 |
| *rrnL* | 12808 | 14091 | - | 1284 | / | / | / | 44 |
| *trnV* | 14136 | 14204 | - | 69 | TAC | / | / | 0 |
| *rrnS* | 14205 | 14968 | - | 764 | / | / | / | 0 |
| AT-rich region | 14969 | 15399 | / | 431 | / | / | / | / |
